# Technology-Assisted Upper Limb Therapy (TAULT): Evaluation of Clinical Practice at a Specialised Centre for Spinal Cord Injury in Switzerland

**DOI:** 10.3390/healthcare11233055

**Published:** 2023-11-28

**Authors:** Daniela B. Kuchen, Beatrice Hubacher, Andris Ladner, Inge-Marie Velstra, Mario Widmer

**Affiliations:** 1 Neuro-Musculoskeletal Functioning and Mobility, Swiss Paraplegic Research, 6207 Nottwil, Switzerland; mario.widmer@paraplegie.ch; 2 Department of Therapy, Swiss Paraplegic Centre, 6207 Nottwil, Switzerland; 3 School of Health Sciences and Medicine, ZHAW Zurich University of Applied Sciences, 8401 Winterthur, Switzerland; 4 Clinical Trial Unit, Swiss Paraplegic Centre, 6207 Nottwil, Switzerland; inge-marie.velstra@ffhs.ch; 5 Department of Business and Technology, FFHS University of Applied Sciences, 3900 Brig, Switzerland

**Keywords:** tetraplegia, robotics, rehabilitation, upper extremity, goal setting

## Abstract

(1) Improving upper limb function is essential for people with tetraplegia. Although promising, technology-assisted upper limb training is understudied in this population. This article describes its implementation in a Swiss spinal cord injury rehabilitation centre and reports on the observed changes. (2) A retrospective evaluation of clinical data from January 2018 to June 2020 examined patient characteristics, training parameters, goal-setting practices, goal achievement, and changes in muscle strength over the course of technology-assisted upper limb training. (3) Data analysis included 61 individuals, 68.9% of whom had a spinal cord injury. The ArmeoSpring was the most frequently used device. The typical treatment regimen was three 25 min sessions per week, with evaluations approximately every six weeks. The 1:1 sessions, delivered by specialised staff, focused primarily on improving shoulder movement and the ability to eat and drink. Functional goals were set using a grid. Performance on selected goals in the areas of ‘body functions’ and ‘activities & participation’ as well as muscle strength, increased over the course of training. (4) The ArmeoSpring has broad applicability. Despite the observed improvements, the isolated effect of technology-assisted upper limb training cannot be concluded due to the lack of a control group and various concurrent interventions.

## 1. Introduction

Tetraplegia describes a health condition in which both arms, both legs, and the trunk are at least partially paralysed. The most common cause of tetraplegia in adults is a cervical spinal cord injury (cSCI), although Guillain-Barré syndrome (GBS), or multiple sclerosis, among others, can also lead to tetraplegia. Due to limited upper limb function, individuals with tetraplegia often rely on support in daily life. Previous studies have shown that restoring upper limb function is one of the most important priorities for people with cSCI [1] and is thought to be important for regaining independence and quality of life.

Repetitive and activity-based exercise has been suggested as a means to facilitate recovery after spinal cord injury (SCI) by inducing practice-dependent brain and spinal plasticity [2]. Technology-assisted training is a promising approach with the potential to save personnel resources and increase the number of repetitions during a therapy session [3]. Moreover, patients are typically engaged in their therapy exercises in a playful manner, motivated by game-like performance feedback in a setting that allows for the control of numerous training variables. Motivation to train can be further increased by setting goals. It is particularly important to involve patients in the goal-setting process, as goals are only effective if patients find them meaningful [4]. In addition, interventions can be guided and progress monitored [4]. In addition to providing a common language and basis for defining and measuring health and disability [5], the ICF framework has been suggested for use in goal setting [6] and for guiding therapeutic interventions in a common, standardised language [7].

Although technology-assisted training is increasingly used in daily clinical practice for the rehabilitation of people with tetraplegia, there is no consensus on which device is most effective, what an optimal training protocol looks like, and at what stage it is most useful [8]. In order to gain in-depth knowledge about the dosage of treatment, Yozbatiran and Francisco [9] emphasise the importance of investigating different training modalities. For an indication of where to start, retrospective data analysis can help to clarify hypotheses and determine an appropriate study design for a prospective study [10].

The Swiss Paraplegic Centre (SPC) is striving to complement standard therapy with new technologies to increase repetitions during the rehabilitation and to give the patients an opportunity to work on their goals in a playful way. The SPC is the largest specialised centre for the rehabilitation of people with SCI in Switzerland and also treats other diseases with similar symptoms (e.g., GBS). Technology-assisted upper limb training (TAULT) at the SPC is administered using devices from the Armeo therapy concept (Hocoma AG, Volketswil, Switzerland). 

There is a growing number of annually published articles featuring the term ‘Armeo’, highlighting the increasing interest in TAULT. Whereas most of these studies have focused on other target populations, only a few articles have described Armeo training for people with tetraplegia.

Therefore, the aim of this study was to retrospectively analyse (I) the current routine of TAULT at the SPC, (II) goal-setting practice, and (III) whether changes in functioning were observed along with TAULT.

## 2. Materials and Methods

### 2.1. Design and Sample

This retrospective analysis included all adults (aged ≥ 18 years) who underwent TAULT during their rehabilitation at the SPC between January 2018 and June 2020. Clinical records and TAULT documentation were used to extract study relevant data. The following demographic information was extracted: diagnosis (for SCI, neurological level of injury classified according to the International Standards for Neurological Classification of SCI (ISNCSCI), expressed by level of injury and ASIA Impairment Scale (AIS) [11]), age, sex, days since injury, and days since admission. All data are rounded to one decimal place.

### 2.2. Technology-Assisted Upper Limb Therapy (TAULT)

Devices: The upper limb robotic park at the SPC consists of three devices: ArmeoPower, ArmeoSpring and ArmeoBoom (Hocoma AG, Volketswil, Switzerland). With the ArmeoPower, the arm of the patient is placed in an exoskeleton structure. The system provides both anti-gravity weight support and adjustable assistance to the patient’s movement. It allows six actuated axes of motion (shoulder flexion (F)/extension (E), shoulder abduction (Abd)/abduction (Add), shoulder internal rotation (IR)/external rotation (ER), elbow F/E, forearm pronation (Pro)/supination (Sup), wrist F/E) with an additional grip module. According to the manufacturer, the ArmeoPower is specifically designed for upper limb therapy in early stages of rehabilitation, allowing patients with severe motor impairments to perform high numbers of repetitions. Its sister product, the ArmeoSpring, works in a similar way but uses springs to counterbalance the weight of the device and the user’s upper limb, and thus has no motors to actively assist the movement. It therefore enables self-initiated arm movement recruiting any remaining upper limb motor function. Lastly, the ArmeoBoom consists of an overhead sling suspension system, which is suitable for patients who can actively move their arm but who suffer from reduced active range of motion (ROM) and poor motor control. Similar to the ArmeoSpring, the ArmeoBoom provides adjustable arm weight support and has no motors to actively guide movements.

Robotic Concept: These devices were used according to an in-house robotics concept introduced by occupational therapists and a robotics specialist. As part of this routine, the case-managing therapist enrolled a patient for TAULT and was responsible for documenting data related to this therapy, such as the diagnosis, choice of device, date of assessments, suggested frequency, and duration of training. The most appropriate device was evaluated by an expert for Armeo therapy and the case-managing therapist, and a block of approximately 6 weeks of training was planned. Training was supervised in a 1:1 setting by an occupational therapist or therapy assistant. An evaluation after each training block determined whether the patient would benefit most from continuing to train with the same Armeo device, changing the Armeo device to suit their level of motor function, or discontinuing TAULT. Evaluation was based on clinical assessments and goal attainment scores.

### 2.3. Goals

According to the ICF framework, two types of goals were set prior to TAULT. Firstly, two goals corresponding to the ‘body functions’ domain and secondly, an Activity of Daily Living (ADL) goal to cover the ‘activities & participation’ domain. To evaluate these goals, a scale inspired by the goal attainment scale (GAS) was used. GAS is a method for assessing the extent to which the patient’s individual goals are achieved during the course of the intervention [12,13]. It is a widely recognised outcome measure for this purpose, offering validity, reliability, and sensitivity [14]. At the SPC, the performances of targeted goals were subjectively rated by the therapists together with the participant on a 9-point scale ranging from −4 to 4 (−4 = 100% worse, −3 = 75% worse, −2 = 50% worse, −1 = 25% worse, 0 = no change, 1 = 25% improvement, 2 = 50% improvement, 3 = 75% improvement, 4 = 100% improvement/goal achieved). This differs from the originally described GAS, where goals are rated on a 5-point scale ranging from −2 (least favourable outcome) to 2 (most favourable outcome) [15]. 

#### 2.3.1. Body Function

The case-managing therapists defined two goals in the ‘body functions’ domain. The definition was facilitated by the use of a scoring grid from which the following options could be selected: (1) shoulder F/E, (2) shoulder Abd/Add, (3) elbow F/E, (4) Pro/Sup, (5) wrist F/E, (6) coordination, (7) grip strength, (8) power dosing in the hand, (9) motor learning, (10) regulation of tone, (11) pain reduction, (12) preservation of function. For (1)–(3), the target component of physical fitness had to be defined. The available options were (a) ROM, (b) strength, and (c) endurance.

#### 2.3.2. ADL Goals 

Moreover, a goal for ADL was defined together with the participant in free-text format. These goals were then assigned to the corresponding ICF category by a research assistant. Using the ICF linking rules [16], they were categorised as precisely as possible, i.e., at the highest possible level in the ICF, and in a second step, consolidated into activities. To further condense the list of goals, they were discussed with researchers with expertise in TAULT and with tetraplegia-specialised occupational therapists. The aim of the discussion was to merge goals with a similar therapeutic approach (e.g., ‘d550 Eating’ and ‘d560 Drinking’). In addition, categories with rare mentions were integrated into other thematically related categories, e.g., ‘d170 Writing’ into ‘d440 Fine Hand Use’. All goals that fit into the ICF domain of ‘body functions’ were grouped into one category, as the intention was to have goals that were related to ADL. Discussion of the allocation and groups was continued until a consensus was reached. Finally, goal attainment scores were analysed for each category.

### 2.4. Manual Muscle Testing

SPC therapists are encouraged to update the muscle status using the Medical Research Council Muscle Grading Scale on a monthly basis. The strength values of 4 muscle groups (biceps brachii, triceps brachii, clavicular part of the deltoid, and acromial part of the deltoid) closest to the TAULT evaluation date were extracted from the patients’ records. Each muscle was scored on a scale from 0 to 5, with 0 representing complete paralysis and 5 representing normal strength [17].

### 2.5. Data Analysis

Data are summarised descriptively, using absolute numbers and percentages or median and interquartile range (IQR), and presented graphically. Demographic and medical context data, device use data, training parameters, and defined functional goals are displayed. Furthermore, we present a descriptive pre-post comparison for goal achievement and muscle strength. To assess potential differences in improvement between different subgroups, data of these groups were analysed separately. In order to present the distribution of diagnoses among the devices, SCI was divided into high tetraplegia (C1–C5) and low tetraplegia (C6–T1) and was further split into motor complete (AIS A–B), AIS C and AIS D. As upper limb muscles are not affected in paraplegia (T2–S3), paraplegic patients from all AIS grades were grouped together, finally resulting in the following SCI groups: (A) C1–C5 AIS A–B; (B) C1–C5 AIS C; (C) C1–C5 AIS D; (D) C6–T1 AIS A–B; (E) C6–T1 AIS C; (F) C6–T1 AIS D; (G) T1–S3 AIS A–D.

## 3. Results

### 3.1. Demographics

Between January 2018 and June 2020, a total of 78 upper limbs of 61 participants (15 female, 46 male) underwent at least one block of 6 weeks of TAULT. Table 1 shows the demographics of the study sample. SCI was the most common diagnosis, followed by GBS. Among individuals with SCI, the number of users decreased with decreasing lesion level. 

### 3.2. Technology-Assisted Upper Limb Therapy (TAULT)

**Figure 1:**
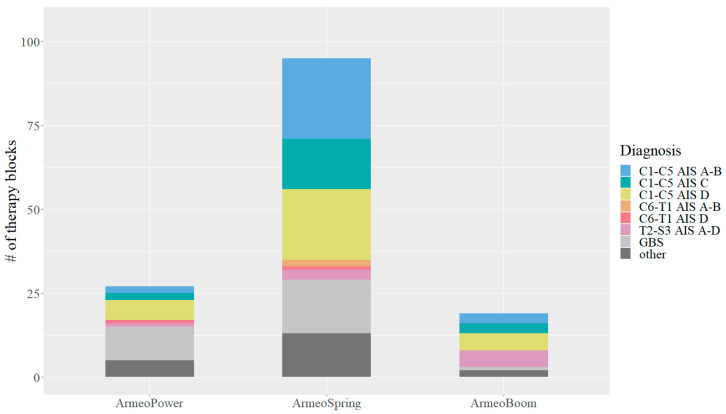
Number of users and distribution of diagnoses across devices. AIS: American Spinal Injury Association Impairment Scale; GBS: Guillain-Barré syndrome; C: cervical, T: thoracic, and S: sacral.

A total of 141 training blocks were recorded, 84 blocks (59.6%) for the right arm and 57 (40.4%) for the left arm. Blocks lasted approximately 6 weeks (median: 6.1 weeks, IQR: 5.4–8.8). The median number of times participants trained with the same device before changing device or discontinuing training was 25 (IQR: 15.8–35.0). Overall, 48.7% and 32.1% of the participants underwent 1 and 2 training blocks of TAULT, respectively. Moreover, 15.4% changed the device after the evaluation of a training block. In total, 89.4% of the participants exercised three times, 2.8% exercised two times, and 2.1% exercised four times per week, while 5.7% had no documented training frequency. Most of the training sessions (81.6%) lasted 25 min, and only 9.9% of the sessions were scheduled for 35 min. For 8.5% of the sessions, the training duration was not recorded. The number of users and the distribution of the different diagnoses across the training devices is shown in Figure 1. Remarkably, the ArmeoSpring was by far the most frequently used device. 

### 3.3. Goals

#### 3.3.1. Body Functions

**Figure 2:**
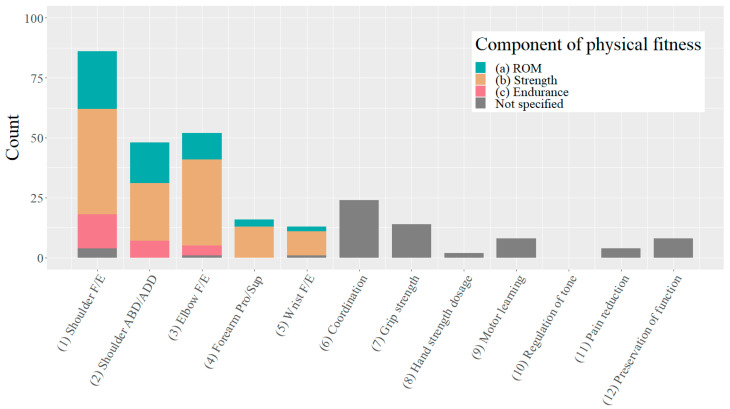
Goals in the ‘body functions’ domain. Two goals were to be selected per participant for each block of ~6 weeks of technology assisted upper limb therapy. ABD/ADD: abduction/adduction; F/E: flexion/extension; Pro/Sup: pronation/supination.

In the ‘body functions’ domain of the ICF, two goals per training block were set by the case-managing therapist using a scoring grid with predefined selection options ((1) to (12)). Hence, 282 goals were evaluated. They mainly aimed at improving the strength to move the shoulder or elbow joint (Figure 2).

The robotics concept also foresees that the addressed component of physical fitness ((a)–(c)) should be defined for objectives (1)–(3). Most of the therapists followed the concept, as there were only a few missing values (i.e., ‘Not specified’) for the goals (1)–(3). Although this was not required by the concept, most even defined the physical fitness component for goals (4) and (5) (Figure 2). 

**Figure 3:**
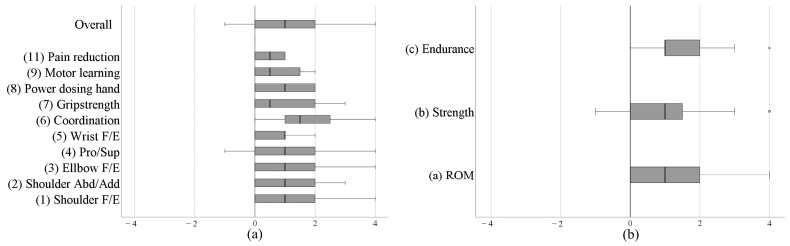
Achievement of goals in the ‘body functions’ domain evaluated on an adapted Goal Attainment Scale ranging from −4 to 4 (100% deterioration—100% improvement in performance of the targeted function); (**a**) ‘body functions’ goals using a grid, (**b**) component of physical fitness Abd/Add: abduction/adduction; F/E: flexion/extension; Pro/Sup: pronation/supination; ROM: Range of motion.

At the end of a training block, 60.4% improved their performance in the target function on the aforementioned nine-point scale, with scores above 0 indicating progress. The majority of the participants were able to perform the target function 25–50% better than at the beginning of the training block. For 34.4% of the goals, no noticeable change was observed. One participant’s performance of the target function worsened by 25%, and data were missing for 5% of the goals (Figure 3a). Furthermore, for goals where the component of physical fitness had to be selected, the changes were predominantly positive (Figure 3b).

#### 3.3.2. ADL Goals

Overall, 151 ADL goals, formulated in free-text format by 61 participants together with their therapists, were analysed. Some participants set more than one goal, for example, if they trained both the right and left arm, or if they had already achieved their goal and defined a new one during TAULT.

**Figure 4:**
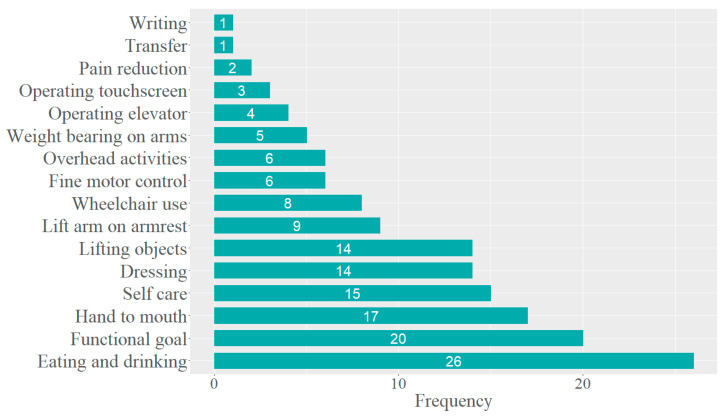
ADL goals formulated in free-text format could be assigned to 16 activities.

**Figure 5:**
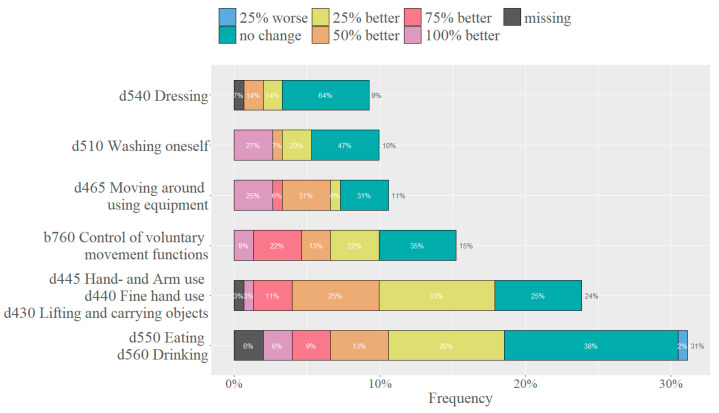
Activities derived from ADL goals formulated in free-text format grouped into 6 categories. Colours indicate a change in the performance of the targeted activity after a therapy block. The number at the end of each bar represents the percentage of that category in all 151 goals.

The goals could be assigned to 16 activities (Figure 4). Through discussion, these activities were then grouped into the following six categories: (i) ‘d550 Eating and d560 Drinking’; (ii) ‘d445 Hand and arm use, d440 Fine hand use, and d430 Lifting and carrying objects’; (iii) ‘b760 Control of voluntary movement functions’; (iv) ‘d465 Moving around using equipment’; (v) ‘d510 Washing oneself’; (vi) ‘d540 Dressing’ (Figure 5). The categories were defined as precisely and closely as possible to the participant’s goals and as broadly as necessary to ensure a manageable and practical number of categories. Specific information on what these items include or exclude can be found in the ICF literature [18].

The most common category was ‘d550 Eating and d560 Drinking’ (31%), followed by the category ‘d445 Hand and arm use, d440 Fine hand use, and d430 Lifting and carrying objects’ (24%). The percentages within a bar indicate how often the different goal achievement scores within the category were mentioned. Overall, 60.2% of the sample reported an improved performance of the targeted activity (between 25% and 100% better). Only one participant reported a worse performance (25% worse) regarding a goal in the category of ‘d550 Eating and d560 Drinking’. Least successful for goal achievement was ‘d540 Dressing’, with two-thirds of the cases reporting no change.

### 3.4. Manual Muscle Testing

**Figure 6:**
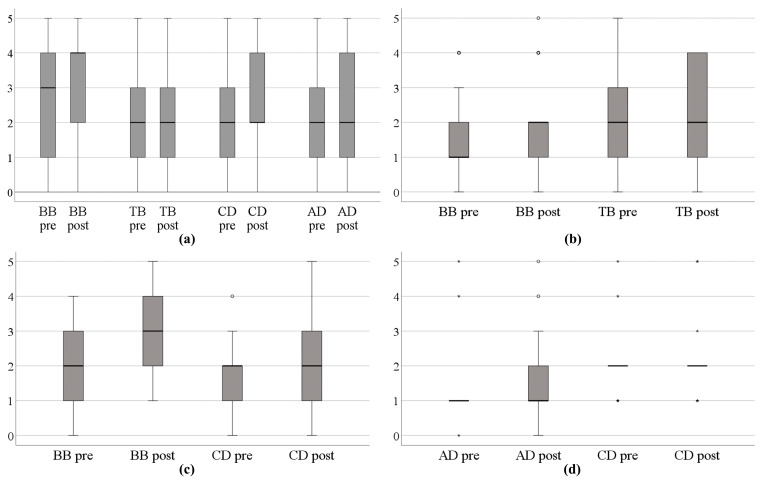
Muscle strength before and after a training block. (**a**) Overall, (**b**–**d**) when the goal in the ‘body functions’ domain was to improve strength in (**b**) elbow flexion/extension, (**c**) shoulder flexion, (**d**) shoulder abduction. BB: biceps brachii; TB: triceps brachii; CD: clavicular part of the deltoid; AD: acromial part of the deltoid.

Routinely tested upper limb muscles showed an increase in muscle strength after a training block (Figure 6a). When the explicit goal was to improve a particular movement direction, there was also a noticeable trend for improvement in muscle strength within the muscles responsible for that movement (Figure 6b–d).

## 4. Discussion

The ArmeoSpring was the most frequently used device at the SPC for TAULT, typically with the goal to improve the strength of the muscles moving the shoulder and elbow joint. Muscle strength increased overall, but particularly in the trained direction. Moreover, 60.4% of the target functions in the ‘body functions’ domain showed improved performance after TAULT. Analysis of ADL goals identified six main categories, with the majority of participants reporting improved performance in the targeted activity following a training block. In the ‘activities & participation’ domain, ‘d550 Eating and d560 Drinking’ was the category to which the greatest number of ADL goals were assigned to. A typical therapy regimen included three weekly sessions of 25 min each over a period of 6 weeks.

### 4.1. Sample

In line with the specialisation of the SPC, the most common diagnosis for participants using the upper limb robotic park at the SPC was SCI (68.9%). According to recent literature, the spinal cord population in higher-income countries is approximately 27% female [19]. The cohort of the present study had 25% female participants overall, but less than 20% of SCI patients were female. With 13.1%, the second most frequent diagnosis was GBS, which is more common in males (the male-to-female ratio is typically 1.5:1 [20]). In conclusion, it is not clear whether there is a difference in referrals for TAULT between the sexes, with males potentially showing more interest in TAULT, or therapists assuming that males are more open to technology.

In order to depict which individuals with SCI were the most frequent users of these devices, the severity of SCI was categorised differently than previously recommended as a reporting standard [21]. The authors felt that this classification might be too broad. A finer grading was used, taking into account several aspects. Firstly, individuals with a lesion at C6 or below had at least an active tenodesis grip, if not active grip function, and therefore had a great potential to improve grip function compared to individuals with higher lesions. Moreover, lesions below T2 were not expected to affect upper limb function. Therefore, lesion levels from C1–C5, C6–T1, and T2–S5 were grouped together. Secondly, AIS A and B were grouped together due to their motor completeness. Finally, C and D were shown separately because of the greater muscle strength present in individuals classified as AIS D.

The majority of the participants with SCI had a tetraplegia (85.7%), and more than half of them had no active grip function. Furthermore, individuals with a motor incomplete SCI were the most frequent users of the upper limb robotic park, and only few individuals with a paraplegia were referred to TAULT. This suggests that therapists consider TAULT to be particularly effective in improving residual function below the lesion and more likely to improve active movement in larger joints, such as the shoulder or elbow, rather than hand function.

Our analysis has shown that six people with paraplegia were also referred to TAULT. This may seem somewhat confusing but may be due to ongoing shoulder complications such as shoulder pain or rotator cuff injuries.

### 4.2. Technology-Assisted Upper Limb Therapy (TAULT)

Typically, a training block lasted 6.1 weeks, with a frequency of three times per week and a duration of 25 min per training session, indicating that therapists generally adhered to in-house guidelines. However, further studies are needed to determine whether these parameters represent the optimal conditions or whether other training variables may lead to more favourable outcomes. Additionally, it is worth considering whether a 1:1 setting is necessary for TAULT, as existing literature suggests that a 2:1 setting is equally effective and may address cost reduction pressures without negatively affecting patient outcomes [22].

The ArmeoSpring was by far the most frequently used device. It can therefore be concluded that for ArmeoPower and ArmeoBoom, the capacity limits are certainly far from being reached and that the majority of patients with limited upper limb function can exercise with the ArmeoSpring.

TAULT was typically initiated within 76–221 days after the onset of the disease and 31–114 days after admission to the SPC. For people with SCI, TAULT was initiated slightly earlier (median: 105 days after injury and 72.5 days after admission). The retrospective analysis does not allow an analysis of the reasons for the delay between admission and the start of TAULT. However, considering that motor recovery in the SCI population is most rapid within the first three months after injury, with a plateau at 12–18 months after injury [23], early initiation of TAULT could have a beneficial effect on upper limb motor recovery. Therefore, initiating TAULT within the first three months after injury seems reasonable in order to capitalise on any newly acquired movement capability. Yet, this requires patients to be able to sit in a wheelchair and have a circulation stable enough to participate in such a training.

### 4.3. Goals

Goal setting and evaluation serve as valuable tools to guide interventions, monitor progress, and increase patient motivation [4] and are key components of rehabilitation. In this context, two distinct perspectives were covered: the therapists, by formulating goals for the ‘body functions’ domain, and the participants, by formulating an ADL goal. The assessment process was equally comprehensive, with both sets of goals evaluated by the same parties. This two-pronged approach not only takes into account the therapeutic perspective but also integrates the patient’s perspective, adding an extra layer of insight. This approach ensures a holistic assessment that highlights both the patient’s experience and the healthcare team’s expertise. The inclusion of different perspectives enriches the evaluation process, providing a fuller understanding of the effectiveness of the intervention.

The GAS is widely recognised and has good psychometric properties [14]. Its goal-setting process has been described as follows [13]: A goal is formulated that is expected to be achievable, ideally compiling the SMART criteria: specific, measurable, achievable, relevant, and timed. This goal represents the value 0 = goal achieved. Furthermore, two levels for ‘less than expected’ (−1 and −2) and two levels for ‘more than expected’ (+1 and +2) have to be precisely formulated, e.g., with adding/removing support, increasing/decreasing quantity or speed from the goal defined as value 0.

The GAS therefore has five precisely formulated stages where success or failure can be assessed with very little personal judgement. The whole process can be time consuming to implement [12], which can be difficult to fit into the limited time resources. Furthermore, it may not be pragmatic for TAULT to go through the whole process in parallel with the overall rehabilitation goal-setting process, which is an integral part of any rehabilitation at the SPC.

At the SPC, goal achievement was evaluated using a nine-point scale ranging from −4 (100% deterioration) to +4 (100% improvement). Our analysis has shown that there were very few missing values (<7%) for the evaluation of goals, suggesting that this procedure is practical and timesaving. However, the new scale leads to variability in goal scoring as it is not possible to accurately measure the percentage of goal achievement and, more importantly, psychometric properties of the GAS outcome measures are not transferable, making the interpretation of goal achievement uncertain. The authors suggest that the rating of goal achievement be changed to the original scale from −2 to 2. The suggestion is that the value 0, the expected outcome, be precisely defined, while the other stages can remain vague: −2 = much less than expected, −1 = less than expected, 1 = more than expected, and 2 = much more than expected. The authors consider this adaptation to be a practical compromise for TAULT that saves time resources and can be used in daily clinical practice without introducing too much personal judgement and variability into the assessment.

#### 4.3.1. Body Functions

Another approach to safe time resources, the SPC has developed a grid for goals setting specifically for TAULT in the ‘body functions’ domain. This approach aims to increase the efficiency of the goal-setting process, while still providing valuable guidance for TAULT. In brief, the grid provides pre-defined options for the selection of goals in the ‘body functions’ domain. Again, as there were very few missing values for set goals (<7%) this suggests that this is a feasible approach for daily clinical practice. 

Using this grid, therapists predominantly selected goals focusing on improving the function of the major upper limb joints, with shoulder F/E being the most frequently selected movement (Figure 2). These functional goals align well with the most frequently reported ADL goals related to eating and drinking (ICF: d550 and d560, respectively), which require shoulder and elbow control. However, it is important to note that also shoulder external rotation has been shown to be critical in regaining ADL function in traumatic brachial plexus palsy [24]. Although the underlying mechanism of paralysis is different, the resulting limitations in ADL are likely to be comparable. Yet the grid does not include an option to select shoulder rotation as a goal because Armeo does not have standard games specifically designed for this movement. Given the fundamental importance of shoulder external rotation for ADLs, it is recommended to include the option to select it as a goal and allow the therapist to determine how to implement it. For instance, specific instruction on how to perform a particular movement could be provided and monitored, particularly in the context of 1:1 therapy sessions. Further research could explore explicit training methods and game programming for shoulder rotation.

The robotic concept entails that for goals (1) to (3) the addressed component of physical fitness to be improved must be defined. This analysis showed that in clinical practice, therapists typically specify this for goals (4) and (5) as well (Figure 2). Considering the nature of goals (1) to (5), they only define a plane of motion of a joint, which is too unspecific for goal setting. Thus, it seems reasonable to adapt the robotic concept and require the definition of physical fitness not only for shoulder and elbow movements, but also for Pro/Sup and wrist F/E movements.

The evaluation of the goals in the ‘body functions’ domain revealed a trend towards improvement in the targeted function following TAULT. Across the selected goals, coordination showed promising results (Figure 3a). Although not yet prospectively validated, this finding is of relevance for future prospective trials using TAULT in similar study populations. Typically, effectiveness of TAULT is evaluated using well established clinical assessments either focusing on strength (e.g., Upper Extremity Motor Score) [12] or upper limb function (e.g., GRASSP for cSCI [25]) [26]. These evaluations will likely fail to detect improvements of upper limb coordination in the shoulder and elbow joint as specifically trained during TAULT. Unfortunately, there is a lack of valid and reliable assessments of upper limb coordination that are feasible for clinical practice. Marker-based motion capture measurements of upper limb movements, the current gold standard, are time- and labour-intensive, requiring expensive camera systems. Moreover, it would be preferable to be able to quantify upper limb kinematics during ADL activities outside of the lab in order to track changes in upper limb coordination in the real world. Thus, for the future, inertial measurement units and (marker-less) camera-based systems may be more affordable and feasible solutions after their clinical validity has been established.

#### 4.3.2. ADL Goals

In clinical practice for TAULT, ADL goals are freely formulated by the participant in collaboration with the responsible therapist. The aim is to define meaningful activities for the patient’s daily life and to cover the ‘activities & participation’ domain of the ICF. In order to find out where the goals mentioned by the sample belong in the ICF structure, these goals were linked to the corresponding ICF categories. However, this analysis revealed that, despite the explicit request to formulate an ADL related goal, a substantial number of the goals were linked to a category in the ‘body functions’ domain. This is unfortunate not only because it implies redundancy with the functional goals set by the therapists, but also because patients lack a clear idea of the area of daily living that TAULT is intended to improve. Therefore, therapists should take sufficient time to guide and facilitate a shared decision-making towards an achievable ADL goal that is important to the patient. To find a way to make this process more efficient, goals were first assigned to an activity (Figure 4), and then activities with a similar therapeutic approach were grouped together (Figure 5). A struggle while working with the ICF framework was to choose the highest, most precise level while still including as much information as possible. Some categories were described by more than one ICF code because a single code would not adequately describe the goals set in that category, while a more general level would include much more than is useful. In the end, six categories remained, one of which summarised all the goals in the ‘body functions’ domain (‘b760 Control of voluntary movement functions’). The other five main categories that emerged from this analysis (Figure 5) represent goals that have been frequently mentioned by patients with tetraplegia and their therapists in the literature [27,28] and by occupational therapy experts at the SPC. As these five categories seem to cover the areas commonly selected as goals by individuals with tetraplegia and their therapists, it is suggested that they be used as a grid, analogous to the goals in the ‘body functions’ domain. The categories will serve as a reminder and basis for discussion for both patients and therapists when setting a goal. This has the potential to simplify the goal-setting process and facilitate ADL goal-oriented therapy for TAULT in daily clinical practice. In addition, ADL goals are not usually evaluated in quantitative studies, although they are highly important and motivating for patients. This approach provides a way to assess ADL goals consistently in future studies.

In terms of goal achievement, the categories ‘d540 Dressing’ and ‘d510 Washing oneself’ contain activities that appear to be difficult to improve (64% and 47% respectively reported no change). The goals in these categories often included movements that require a large ROM, such as moving the arm overhead or behind the back. This suggests that activities requiring a wide ROM are difficult to regain and may be difficult to train with the Armeo devices. However, further research is needed to confirm this assumption.

### 4.4. Manual Muscle Testing

Overall, when descriptively evaluated, an increase in muscle strength was observed after TAULT, except for the triceps (Figure 6a). When the explicit aim was to increase strength in a specific plane of movement, the agonist muscles also showed a general trend of improvement (Figure 6b–d). First of all, it should be noted that these changes were not statistically validated and that the participants were primarily individuals in the sub-acute phase following a SCI during their inpatient rehabilitation stay. This rehabilitation phase is characterised by a natural neuronal recovery that usually occurs by the ninth month after injury [23]. Additionally, alongside TAULT, the participants received a variety of therapeutic interventions during their rehabilitation. Therefore, any findings of change in this study should be interpreted with caution. To disentangle different factors (neuronal recovery, the effect of TAULT, other rehabilitative interventions), a prospective study under controlled conditions would be needed. Furthermore, there is an ongoing debate about how to measure muscle strength in clinical practice and research. Whereas manual muscle testing is easy to use and requires no equipment, it demands a highly standardised procedure and an experienced assessor to ensure reliability [29]. Nevertheless, it is not very responsive, and its reliability is limited, particularly for scores above 3 [30], where, in our opinion, a considerable degree of subjective judgement by the assessor is involved. An alternative approach is to use a handheld dynamometer. Handheld dynamometry is reliable, and due to its high resolution, it is capable of accurately measuring even subtle changes in muscle strength. However, similar concerns about standardised testing procedures to those associated with manual muscle testing remain [31]. Moreover, this measurement method may be less practical for the spontaneous muscle strength measurements often conducted by therapists in their daily clinical practice, as it requires the dynamometer to be carried around constantly in their daily work routine. In conclusion, for a prospective study, the use of a reliable and responsive method with a high resolution to assess changes in muscle strength, such as handheld dynamometry, is recommended. 

### 4.5. Personnel

Finally, this study highlights the value of having specialised staff dedicated to TAULT. Specialists trained in this area have in-depth knowledge of how to use these devices. This reduces the barrier to referring patients for TAULT, as only specialised staff will be responsible for these treatments, rather than the entire therapy team being expected to have comprehensive knowledge of all technologies and therapy methods. Therefore, this approach has the potential to increase the frequency and quality of TAULT.

In addition, the involvement of specialised staff facilitates consistent data collection. As seen in this study, the low incidence of missing data reflects the rigorous data collection process implemented by a dedicated team.

However, it is important to acknowledge the limitations of this study. In addition to the previously mentioned limitations, the inclusion of predominantly individuals with SCI from one single rehabilitation centre in Switzerland limits the generalisability of the findings to other institutions and other diagnostic groups. Additionally, although this study shows improvements in patients who received TAULT, the lack of a control group prevents conclusions regarding the effectiveness of TAULT due to various parallel interventions and because natural biological recovery cannot be controlled for. Further well-controlled studies are needed to provide more robust evidence and to determine the effectiveness of TAULT in improving patient outcomes. Importantly, these studies should also include a detailed description of the training programme to tackle the “black box” that is often a problem when investigating of a therapy method.

## 5. Conclusions

In summary, this study found that the ArmeoSpring was the most frequently used device in a cohort mostly consisting of individuals with cSCI, underlining its broad applicability for similar populations. Most participants trained three times a week for 25 min over a period of six weeks. The use of a scoring grid to set functional goals proved to be practical and timesaving in clinical practice. In addition, the proposed implementation of an ADL goal-setting grid could facilitate consistent evaluation and enhance goal-oriented therapy for TAULT. Furthermore, specialised TAULT staff are important to ensure consistent data collection and quality of treatment. Over the course of TAULT, targeted functions and activities, as well as strength, have improved. However, due to the absence of a control group, concomitant spontaneous neurological recovery and the concurrent application of various rehabilitative interventions, the effect of the TAULT itself cannot be estimated. Well-controlled trials are needed to provide evidence for TAULT in people with tetraplegia.

## Figures and Tables

**Table 1:** Characteristics of study cohort.

Characteristics	SCI	GBS	Other	Total
Number (%)	42 (68.9)	8 (13.1)	11 (18.0)	61 (100)
Age: median (IQR) (years)	58.0 (47.0–66.3)	70.0 (63.5–77.3)	43.0 (27.5–60.5)	58.0 (45.0–69.0)
Time since disease onset: median (IQR) (days)	105 (70.8–137.5)	162 (114.8–188.8)	222 (81–2181)	118 (76–221)
Time since admission: median (IQR) (days)	72.5 (35.3–106.3)	106 (67.0–144.8)	47 (17.5–119.0)	73 (31–114)
SCI group, *n* (%)	42 (100)			
C1–C5 AIS A–B	13 (30.6)			
C1–C5 AIS C	7 (16.7)			
C1–C5 AIS D	13 (30.6)			
C6–T1 AIS A–B	1 (2.4)			
C6–T1 AIS C	0 (0)			
C6–T1 AIS D	4 (9.5)			
T2–S3 AIS A–D	6 (14.3)			

AIS: American Spinal Injury Association Impairment Scale; GBS: Guillain-Barré Syndrome; IQR: interquartile range; SCI: spinal cord injury; C: cervical, T: thoracic, and S: sacral.

## Data Availability

The datasets generated and/or analysed during the current study are available from the corresponding author on reasonable request.
